# The Evolution of TMR Utilization: A 23-Year Journey from Exploratory Upper Extremity Prosthesis Control to Standard of Care for Major Amputations and Pain Management

**DOI:** 10.3390/jcm15207874

**Published:** 2026-10-13

**Authors:** Matthew D. Ramsey, Jason Zhang, Muhammad T. Hassan, Sophia Bonfigli, Jason H. Ko, Levi J. Hargrove

**Affiliations:** 1 Division of Plastic and Reconstructive Surgery, Department of Surgery, Northwestern University Feinberg School of Medicine, Chicago, IL 60611, USA; matthew.ramsey@nm.org (M.D.R.); jason.ko@nm.org (J.H.K.); 2 Department of Orthopedics, Northwestern University Feinberg School of Medicine, Chicago, IL 60611, USA; 3 Shirley Ryan AbilityLab, Center for Bionic Medicine, 355 E Erie St, Chicago, IL 60611, USA

**Keywords:** targeted, reinnervation, neuroma, acute, delayed

## Abstract

**Background**: Targeted muscle reinnervation (TMR) has become increasingly utilized beyond advanced myoelectric control to treat and prevent neuropathic pain. This study aims to evaluate changes in practice patterns in TMR utilization. **Methods**: A single-center, retrospective review was conducted for TMR procedures from 2002 to 2025 using CPT codes and publications. Case volumes were analyzed by major amputation level, timing, and for patients without amputation. A bibliographic analysis from 2004 to 2025 was then conducted to model corresponding research activity. Trends were analyzed using negative binomial regression and logistic regression with α = 0.05. **Results**: A total of 569 TMR cases were analyzed, including 145 below-knee amputations, 72 above-knee, 102 major upper extremity amputations, and 159 cases for chronic pain in non-amputees. For all-cause indications, there were 196 acute and 373 delayed procedures. Annual TMR volume increased most rapidly from 2016 to 2019, corresponding to new chronic pain applications. Lower extremity cases became and remained more prevalent in 2017, and acute cases became more common in 2025. The proportion of acute TMR cases increased 20% per year (*p* < 0.001), and the odds of receiving acute TMR for a major amputation increased by 17% per year (*p* < 0.001). When stratifying by timing for amputation-related cases only, all-cause, limb-specific reoperation was more frequent after acute TMR versus delayed (33/124 (26.6%) vs. 31/192 (16.1%); *p* = 0.031) with no difference in nerve-specific reoperation (*p* = 0.25). Notably, on average, only 15% of major amputations received acute TMR from 2021 to 2025. Publishing activity for TMR most rapidly increased from 2018 to 2020. **Conclusions**: TMR experienced a rapid evolution in both technique and indications through the late 2010s, mirrored by the published literature. Buy-in from referring surgeons is critical to performing acute TMR, which has been demonstrated to have superior outcomes, but careful consideration must be given to mitigate surgical site complications in this population.

## 1. Introduction

Each year in the United States, almost 200,000 limb amputations are performed for a variety of traumatic, oncologic, and vascular indications [1,2]. Consequently, an estimated 2 million individuals in the U.S. live with limb loss [3]. For this patient population, residual limb pain and phantom limb pain represent significant sources of morbidity, affecting patients’ physical, mental, and social health, with estimates of prevalence ranging from 50% up to as high as 90% [4,5,6,7]. Post-amputation pain also represents a significant contributor to prosthesis abandonment and inability to ambulate postoperatively [8].

Since its first human application in 2002 as a novel method for establishing advanced, intuitive myoelectric prosthesis control, targeted muscle reinnervation (TMR) has spread globally for all major amputation levels. Its dissemination increased after it was serendipitously discovered to have utility for treating neuropathic pain [9]. A retrospective review by Souza et al. in 2014 [10] reported findings that nearly all patients undergoing TMR had resolution of preoperative neuroma pain with no postoperative neuroma formation [10]. These findings were further confirmed by Dumanian et al. in 2019, who demonstrated similar improvements in phantom limb pain and residual limb pain through a randomized clinical trial [11]. Today, TMR has been broadly adopted to treat or prevent post-amputation pain, neuroma formation, and neuropathic pain in patients without major limb loss [12,13,14,15].

Despite its global adoption, however, there remains a paucity of literature on exact case volumes and practice patterns. In this study, we conducted a single institution review of TMR procedures from the first-ever case performed in 2002 through 2025. In particular, we aim to analyze trends in TMR timing (acute vs. delayed), distribution of upper vs. lower extremity amputation, use for neuropathic pain in patients with intact limbs, and changes in case volume.

## 2. Materials and Methods

This study received Institutional Review Board approval prior to the initiation of any data collection. A retrospective, single-institution review was conducted for all TMR cases from 2002 to 2025. TMR procedures were identified using the non-specific CPT code 64905 (nerve pedicle transfer; first stage) associated with a single site of a tertiary academic medical center (ensuring satellite facilities were excluded from the query), and cases were then manually filtered from these procedures. This primarily entailed excluding motor nerve transfers for peripheral nerve injuries. The resulting cases were also reviewed to ensure that multiple nerve transfers and uses of the CPT code 64905 during a single operation did not result in duplicates.

Annual and cumulative case volumes of TMR procedures were then analyzed by major amputation level (defined as proximal to the wrist or ankle), timing (acute vs. delayed), and for other non-amputation indications such as neuropathic pain (upper extremity, lower extremity, trunk). Acute TMR was defined as occurring during the same operation that a major amputation is performed as identified through review of operative reports.

All-cause, limb-specific reoperation was then assessed for the 2012–2025-time interval, and reoperations were further stratified into the following categories: infection, non-healing wound/ulcer, wound dehiscence, hematoma/seroma, soft tissue/amputation revision, and nerve-related procedures. Infection, non-healing wound/ulcer, wound dehiscence, and hematoma/seroma were further grouped as “any wound-related reoperation” to differentiate from nerve-specific and soft-tissue-only revisions without wounds. Many reoperations fell under multiple categories, so each reoperation category was analyzed as a separate binary outcome, with *p*-values adjusted accordingly using the Holm method. The median (IQR) number of reoperations per reoperated patient is also reported and compared using the Wilcoxon rank-sum test.

Major amputation procedures were then identified in a similar manner through CPT codes (Supplemental Table S1) for yearly volume of shoulder disarticulation, transhumeral, transradial, below-knee amputation (BKA), above-knee amputation (AKA), and hip disarticulation cases. These data were used to calculate and track the percentage of patients who receive acute TMR over time, as well as the ratio of upper extremity to lower extremity amputations.

### 2.1. Bibliographic Analysis

A bibliographic analysis was also performed to characterize publishing trends and research activity associated with TMR utilization for the years 2004 to 2025, corresponding to the first publication on TMR in the literature [9]. Comprehensive PubMed queries were constructed for the following domains: total TMR publications, TMR and amputation, TMR and pain, TMR and prophylaxis/timing (acute vs. delayed), and TMR for patients with intact limbs. Search queries are listed in Supplemental Table S2.

### 2.2. Statistical Analysis

Trends over time were evaluated using negative binomial regression for data involving counts and binomial logistic regression for data involving proportions [16,17]. Negative binomial regression results were reported as an incidence rate ratio (IRR) with corresponding 95% confidence interval (CI), representing a percent change in the outcome count data per year. Logistic regression results were reported as an odds ratio (OR) with 95% CI, corresponding to a change in odds of a particular outcome per year. Reoperation rates were compared using Fisher’s exact test and the Wilcoxon rank-sum test. Analysis was performed in R version 4.3.0 using the “ggplot2” and “MASS” packages [18]. Statistical significance was evaluated at α = 0.05.

## 3. Results

A total of 828 unique procedures logged the CPT code 64905, with the first instance of its use appearing in 2012. After exclusion of non-TMR procedures, 552 unique TMR cases were identified and included for analysis from the CPT code search. An additional 17 cases were identified from the Souza et al. 2014 [10] publication in which they state that from 2002 to 2012 at our institution, eight patients with transhumeral amputation and nine patients with shoulder disarticulation had been treated in total. Therefore, our total institutional TMR case volume from 2002 to 2025 is 569. Note that all figures plot CPT code data only and therefore begin in 2012.

These 569 cases include 196 acute and 373 delayed cases and 145 BKAs, 72 AKAs, 14 hip disarticulations, 37 transradial amputations, 41 transhumeral amputations, and 24 shoulder disarticulations. Additionally, there were 133 TMR cases performed for chronic lower extremity pain and 26 cases for chronic upper extremity pain. The remaining cases were performed after non-extremity neuroma excision to treat neuropathic pain originating from the intercostal, occipital, ilioinguinal, iliohypogastric, or genitofemoral nerves.

Annual and cumulative case volumes for TMR procedures are presented wholistically (Figure 1A,B) and then stratified by major amputation level (Figure 1C,D), timing of the procedure (Figure 2A,B), and use for neuropathic pain (Figure 2C,D). The overall TMR case volume increased significantly over the study period. While 17 total TMR cases were performed over the first decade (2002–2012), CPT code usage increased from one case in 2012 to 64 cases in 2025. The most rapid growth in annual cases can be seen occurring from 2016 to 2019, with a pandemic-associated plateau in volume the subsequent year, followed by minor fluctuations in volume over the subsequent five years. On average, from 2021 to 2025, there are 64 cases of TMR performed per year at our single institution (Figure 1A).

TMR utilization for the four most common major amputation levels is also presented in Figure 1C,D. A similar pattern can be appreciated over the same time course with a more nuanced understanding of the specific applications. Upper extremity cases can be seen to be more prevalent until the 2017–2018 transition (Figure 1D), at which point BKAs account for the most cases, corresponding to the most common amputation level in the US. Prior to 2015, less than five total cases of TMR were performed for lower extremity amputations of any level. Interestingly, the annual volume of transhumeral and transradial cases remained approximately stable over the last decade (Figure 1C).

Case volume plotted by timing of TMR intervention and for specific neuropathic pain applications is displayed in Figure 2. Acute TMR can be seen to increase from one case per year from 2012 to 2017 to 27 cases per year on average over the last 5 years of the study period, 2021–2025. Notably, delayed cases can be appreciated to approximately plateau in annual volume from 2019 to 2021 and then downtrend through 2025, with acute cases becoming more prevalent than delayed cases for the first time in 2025 (Figure 2A). TMR for neuropathic pain in patients with intact limbs can be seen to increase starting in 2017, with rapid growth through 2020–2021 and then an approximate plateau in annual volume through the end of the study period.

Secondary analyses on TMR volume were then performed. The proportion of acute TMR cases compared to total TMR cases increased significantly from 2012 to 2025, with an estimated 20% increase per year (IRR (95% CI) = 1.20 (1.12, 1.28), *p* value < 0.001) (Figure 3A). The ratio of TMR for major lower extremity amputations relative to the upper extremity also increased by an estimated 24% per year (IRR (95% CI) = 1.24 (1.14, 1.35), *p* value < 0.001) (Figure 3B). Lower extremity volume first became more prevalent in 2017 and remained so through the end of the study period. On average over the last 5 years, lower extremity case volume is five times higher than upper extremity case volume.

The yearly volume of major amputations also increased across the study period from 4 to 125 (Figure 4A). For these major amputations, the proportion of cases receiving acute TMR also increased across the study period, corresponding to a 17% increase in odds of receiving acute TMR for a major amputation per year (OR (95% CI) = 1.17 (1.11, 1.24), *p* value < 0.001) (Figure 4B). When stratifying by extremity, there was an 18% increase in odds of receiving acute TMR for major lower extremity amputation (OR (95% CI) = 1.18 (1.11, 1.26), *p* value < 0.001) per year and a 13% increase in odds of receiving acute TMR for major upper extremity amputation, though this latter value did not reach significance (OR (95% CI) = 1.13 (0.96, 1.35), *p* value = 0.150). Of note, from 2021 to 2025, 85% (442/523) of BKAs, AKAs, transradial, and transhumeral amputations did not receive acute TMR. Of the four levels listed, transhumeral was the lowest volume but most likely to receive acute TMR, and AKA was the second most common by volume but least likely to receive acute TMR.

### 3.1. Reoperation Rate

For patients receiving TMR for major amputations, 64/316 cases (20.3%) underwent limb-specific, all-cause reoperation, and 31 (9.8%) had a nerve-related reoperation (Table 1). The additional 17 cases identified from Souza et al. were excluded from the reoperation analysis due to lack of available data. When stratifying by timing, all-cause reoperation was more frequent after acute TMR versus delayed (33/124 (26.6%) vs. 31/192 (16.1%); *p* value = 0.031). The median (IQR) number of reoperations per reoperated patient was 1 (1–3) after acute and 1 (1–2) after delayed TMR (*p* value = 0.073).

When stratifying by reoperation type, patients who receive acute TMR had higher rates of wound-related reoperation (*p* < 0.001) and soft tissue/amputation revision procedures (*p* = 0.001), with no difference in nerve-specific reoperation compared to delayed TMR (*p* = 0.250) over the entire 2012–2025 period (Table 1). A sensitivity analysis of reoperation rate was also performed by time period (2012–2014, 2015–2020, 2021–2025). All-cause limb-specific reoperation was higher in acute TMR patients than delayed TMR for both 2015–2020 (33.3% vs. 21.5%; *p* value = 0.20) and 2021–2025 (23.5% vs. 10.1%; *p* value = 0.023), while only the 2021–2025 period reached significance. Trends over time for reoperation rates following TMR for major amputation were also assessed (Figure 5A). There was no significant change over time in all-cause, limb-specific reoperation or nerve-related reoperations. For TMR in patients with intact limbs, 32 of 236 cases (13.6%) had a nerve-related reoperation (Figure 5B).

### 3.2. Bibliographic Analysis

Publishing activity in all domains of TMR research increased significantly. The number of overall TMR publications increased by 22% per year (IRR (95% CI) = 1.22 (1.19, 1.24)) over the course of the study period. Figure 6A is also annotated with major publication milestones such as the first reference to pain management in the setting of major limb loss, first non-amputation publication, and first review. For TMR associated with amputation, there was an estimated 20% increase in related publications per year (IRR (95% CI) = 1.20 (1.18, 1.23), *p* value < 0.001) (not plotted). For TMR as a surgical treatment for pain, there was an estimated 40% increase in related publications per year (IRR (95% CI) = 1.40 (1.33, 1.47), *p* value < 0.001) (Figure 6B). Similarly, for TMR and prophylaxis/timing and TMR use in neuromas not associated with major limb loss, there were annual increases of 50% (IRR (95% CI) = 1.50 (1.37, 1.68), *p* value < 0.001) and 52% (IRR (95% CI) = 1.52 (1.16, 2.46), *p* value = 0.015), respectively (Figure 6C,D).

## 4. Discussion

### 4.1. Trends in TMR Timing and Utilization

Our institutional sample of 569 TMR patients from 2002 to 2025 demonstrates numerous clinically relevant trends in TMR, from overall increased utilization for major limb loss to atypical neuroma excision and nerve transfer, that are also reflected in national and global trends as TMR is increasingly adopted [13,19]. As an innovator and initial advocate for TMR, our institution can serve as a model for studying how utilization of the procedure has evolved, particularly with regard to nuances in technique by plotting expanding indications through time. Notably, the increase in our institutional case volume precedes the rapid increase in TMR publishing activity by several years, with our volume most rapidly expanding from 2016 to 2019. Other highly relevant time points are (1) lower extremity cases becoming and remaining more prevalent since 2017, with BKAs accounting for approximately 57% of our institution’s major amputations and 54% of amputation-related TMR cases over the last 5 years; (2) neuropathic pain applications rapidly expanding regardless of limb loss since 2017, with now approximately 47% of all TMR cases being performed to treat or prevent neuropathic pain in patients with intact limbs over the last 5 years; (3) acute cases rapidly expanding after 2016, while delayed cases began to down trend since 2022, with acute cases becoming more prevalent than delayed cases in 2025 for the first time; and (4) despite our institution pioneering TMR, just 15% of all major amputations received acute TMR over the last 5 years.

The increase in cumulative TMR case volume accelerated over the most recent five years, likely associated with publication milestones such as the 2019 randomized clinical trial demonstrating the efficacy of TMR for reducing phantom limb pain in patients with major limb loss [11]. A 2014 study from our institution described 17 patients—eight patients with transhumeral limb loss and nine patients with shoulder disarticulations—receiving TMR for advanced myoelectric prosthesis control from 2002 to 2012. This number has since increased to over 300 TMR procedures in the last five years alone, exemplifying the explosive growth of TMR utilization within our surgical center [10]. This trend, however, must also be interpreted within the context of significantly increasing major amputations in the US healthcare system, which increases the potential patient population for which acute TMR may be applied [20,21,22]. Systematic reviews published within a similar time period report samples of 449 cases up to and including 2021 [23] and 778 cases from 2010 to 2023 [24]. Our single site’s volume appears to be comparable to total utilization across these studies, supporting the use of our data to extrapolate more global trends in TMR application while allowing for more nuanced statistics to be plotted.

Transient fluctuations in case volume were observed during and around 2020, likely corresponding to healthcare disruptions amidst the COVID-19 pandemic that delayed many non-emergent surgical procedures [25]. Despite this, however, overall trends were robust across the study period. Our observed trend in lower extremity cases quickly becoming more prevalent than upper extremity cases is corroborated by the TMR literature and well-known amputation statistics. A recent 2024 study analyzed US national data to conclude that nearly 91% of patients living with limb loss suffer lower extremity amputation secondary to vascular disease and/or diabetes, both of which are projected to increase by 36% and 67%, respectively, by 2060 [20]. This same study, as well as two additional recent publications in 2025 and 2026, corroborate the sharp uptrend in amputation rates we present for our institution in Figure 4A [21,22]. All three publications display a rapidly increasing change in amputation rates from approximately 2010 to 2023 which overlaps with our study period. Most compelling is the study by Reilly et al. which found a 65% increase from 2016 to 2023 in lower extremity amputations in Illinois specifically secondary to diabetes and vascular complications which very strongly supports the trend in Figure 4A. And among major lower extremity amputations, BKAs remain the most prevalent [26,27,28,29]. Furthermore, a systematic review on TMR by ElAbd et al. demonstrated that the majority (66%) of patients receiving TMR had lower-limb amputations, of which the most common group was BKA [23].

This trend represents a deeper paradigm shift in the surgical application of TMR, as the majority of these patients with lower-limb amputations will not be fit with myoelectric prostheses—the original intended use of TMR. Rather, this trend is a byproduct of the discovery of TMR to treat neuropathic pain and reduce symptomatic, postoperative neuroma formation, thereby allowing TMR case volume to simply reflect relative frequencies of amputation levels in the US. Although, importantly, these patients still have the full potential to be fitted for a myoelectric prosthesis should the opportunity arise. Along this same time course and as a direct result of TMR’s pain efficacy, our data also demonstrates the increased use of TMR for patients with intact limbs as described by multiple publications from our institution [30,31,32,33]. This application now accounts for nearly half of all TMR performed at our single site.

Our observed pattern in acute TMR volume also predates and is reflected in the literature, as several studies have since been published (2019, 2024) demonstrating acute nerve transfer to be superior to delayed TMR for management of post-amputation pain [12,34,35]. With just one TMR CPT code logged in 2012, the plotted ratio of 1.0 is skewed and not representative of true care trends. Excluding this, there were still some fluctuations in the ratio of major amputations receiving acute TMR from 2012 to 2018, which may be the result of the smaller sample size of major amputation procedures logged during this time. The overall trend of increasing application of acute TMR likely reflects both an increased number of surgeons trained to perform TMR and increased education and buy-in from referring surgeons performing the primary amputation.

However, surprisingly, TMR still remains largely underutilized, with about 85% of major amputations performed over the last 5 years not receiving acute TMR for pain prophylaxis. While we do not advocate for a 100% acute intervention rate, it is the published consensus that if the amputation site of a clinically stable patient without other neurological disorders is judged to be clean enough for wound closure, and if sensation of the residual limb is grossly intact, the patient is a candidate for acute TMR [36]. By these criteria, one would expect more than 15% of patients to be candidates, even in the presence of peripheral vascular disease and advanced diabetes mellitus. Beyond interdisciplinary collaboration and case coordination, more effective characterization of patient-specific risk factors for developing post-amputation pain is needed. This will allow surgeons to effectively target high-risk patients and focus collaborative efforts on a smaller patient population with more to gain from acute TMR. We also recognize that our strict definition of acute, defined as TMR being performed within the same operation as the primary amputation, may also skew this statistic. Finally, there remained a significant proportion of delayed TMR procedures throughout the study period, with over 60% of TMR procedures in our sample being performed after the index operation. A contributor to this is referral bias, as many of the patients receiving delayed TMR are likely being referred from outside institutions for management of post-amputation neuromas, since our institution is a large tertiary regional center with several surgeons experienced in TMR [37]. This, conversely, also likely accounts for the decline in delayed cases, as an increasing number of plastic and orthopedic surgeons are offering TMR at outside institutions, obviating the need to refer.

### 4.2. Reoperation Rates for TMR

Over the 2012–2025 study period, approximately one in five patients undergoing TMR for major amputation required a reoperation on the same limb. Acute TMR was associated with higher all-cause, limb-specific reoperation, but the difference was largely attributable to differences in wound-related complications and soft tissue/amputation revisions, rather than nerve-related procedures. It is unknown if this is directly attributable to the increased dissection needed to perform acute TMR given the inherent decision-making bias discussed below in patient selection, potential increase in tourniquet times, and multiple surgical teams involved in amputation care. In addition, our population is primarily composed of non-traumatic, and frequently dysvascular, patients receiving amputation (Table 1) where the burden of wound-related complications in the early amputation period is expected to be significant. Therefore, simply comparing acute to delayed reoperation rates by no means indicates causality without comparing to amputation complications without acute TMR.

Perhaps most interesting and compelling that yet unidentified factors are critical determinants in this statistic is the fact that no difference was found in all-cause reoperation rates between the upper and lower extremity. One would expect the lower extremity cohort to retain its significance in wound-related complications if the increased dissection required to perform acute TMR in patients with vascular comorbidities is the primary determinant. We do not believe this is an issue of sample size, as the 2021–2025-time interval found significance with a sample of 170 patients while the total lower extremity cohort contains 231 patients. Prospective studies collecting detailed perioperative data are needed to delineate what the true driving factors are behind potentially increased wound-related complications in acute TMR.

For nerve-specific reoperation rates, there was no difference between acute and delayed cohorts. The acute TMR subgroup actually had a lower (though non-significant) proportion of nerve-related reoperations compared to delayed TMR patients, which supports prior work showing reduced symptomatic neuroma formation in prophylactic TMR [35,38]. It is important, however, not to draw conclusions regarding subjective pain outcomes from this statistic.

Finally, in the most recent 5 years analyzed, reoperation rates can be appreciated in Figure 5 to remain roughly stable for acute TMR while they appear to decrease for delayed TMR and for patients who have not suffered limb loss. We believe this may be secondary to increasing experience with preoperative patient selection and decision-making rather than changes to the technical success of the nerve transfer itself. As experience was gained, patient evaluation for candidacy, especially in patients with intact limbs, and surgical planning to comprehensively treat neuropathic pain likely increased proportionally. Conversely, the approximate plateau in reoperation rates after acute TMR is performed is likely due to the unpredictable nature of postoperative neuropathic pain after limb loss and that only “high-risk” nerves such as the superficial peroneal, tibial, and sural nerves are classically transferred in BKAs, rather than exhaustive transfers for a given level of limb loss.

### 4.3. Clinical Decision-Making

While there are no scoring systems or decision-making tools that currently exist to assist with objectively assessing patient candidacy for acute TMR akin to anticoagulation scoring with the Caprini, a recent 2023 consensus article by leading peripheral nerve surgeons in the field proposed patient selection criteria [36]. The consensus proposed that clinically stable patients who (1) are sensate at the planned level of amputation without confounding neurological diagnoses, (2) retain viable, innervated muscle targets in the residual limb, and (3) whose wounds are judged to be clean enough for definite closure if present, can be considered for acute TMR [36]. Prior work has also established younger age, psychiatric diagnoses, preoperative chronic pain, and more proximal amputation levels as risk factors for post-amputation pain, with traumatic etiologies also raising concern [39,40].

Risks and benefits of acute TMR have been primarily debated for dysvascular patients, where the increased dissection needed to perform acute TMR can put tenuous tissue at further risk of malperfusion and incisional necrosis or delayed wound healing. Intraoperative caution is recommended in this patient population given that prior work has established the benefit for performing TMR in highly comorbid patient populations such as this with no statistical differences in complication rates [38,41,42]. In these small, single-center, retrospective cohorts, delayed wound healing is the most common complication, while systematic reviews have found recurrent neuromas to be the most common indication for reoperation [13,43]. Patients deemed poor candidates for acute TMR should be followed to evaluate for residual or phantom limb pain and re-assess candidacy for delayed TMR.

Limitations of both the prior literature and our work include significant implicit decision-making that cannot be quantified, the required presence and availability of a surgeon trained in TMR, and case coordination between specialties. At our institution, collaboration is strongest between plastic surgeons performing TMR, of which there was only one prior to 2016, and vascular or orthopedic surgeons performing the primary amputation. Plastic or orthopedic surgeons comfortable with nerve transfers can easily perform TMR without specialized training, and no microsurgical experience is required. Scheduled outpatient cases with an expected need for TMR are now coordinated with predictable surgeon availability, while unscheduled patients with an indication for TMR can be more difficult to coordinate. An open, interdisciplinary dialog between surgical teams must prioritize patient stability and overall course, clinical urgency to amputate or close an open amputation site, and surgeon availability against the expected outcome for the patient if acute TMR is not performed. Flexibility and a willingness to collaborate for direct patient benefit is mandatory for all providers involved. It is also important to note that this collaboration extends beyond amputation care, primarily with our orthopedic colleagues. Care is also coordinated for patients with neuropathic pain from prior surgeries or trauma or in oncologic resections mandating peripheral nerve sacrifice. These patients also benefit from prophylactic TMR.

Expected patient benefit falls into two categories—chronic pain relief or prophylaxis and enhanced, intuitive control of myoelectric prostheses with multiple, simultaneous degrees of freedom. For advanced myoelectric control, TMR is critical for upper extremity amputation proximal to the elbow, as prosthesis control is dependent on elbow, wrist and hand signals which require nerve transfers to be distinctly generated at recordable amplitudes. At the transradial level, TMR is beneficial but not mandatory for myoelectric use given the significant retention of extrinsic hand musculature in the forearm and lack of prostheses able to replicate intrinsic motor control beyond finger flexion and extension [44]. For major lower extremity amputations, myoelectric prostheses currently only exist in laboratory and research settings, making the primary patient benefit pain relief during conventional, socket-based prosthesis use. This landscape is rapidly evolving, however, so robust and correct nerve transfers for this patient population potentially allow for future myoelectric use without surgical revision.

### 4.4. Research Activity for TMR

The increase in volume of TMR procedures from our institutional data was mirrored by the bibliographic analysis demonstrating increased publishing activity for TMR for its uses in amputation, pain management and prophylaxis and in the treatment of neuromas not associated with limb loss. Our data showed marked increases in TMR case volume starting in 2016, in contrast to the bibliographic analysis which demonstrated increasing research activity for TMR around 2018, suggesting that our institution was an early adopter of TMR with experience preceding that of the national cohort. These trends demonstrate the importance of research for the adoption of new surgical techniques, including both technical descriptions and outcomes, for referring surgeons and for training peripheral nerve surgeons currently in practice.

### 4.5. Translation of Clinical Research to Insurance Coverage

The adoption of a novel surgical technique like TMR requires the accumulation of evidence on its efficacy, but translating these research activities into insurance coverage represents another major hurdle prior to widespread implementation. Insurers must first determine if a new technique meets criteria for coverage, which the Centers for Medicare and Medicaid Services (CMS) defines as being both “reasonable and necessary” [45]. Afterwards, billing codes must be created or amended to include the procedure through governing bodies such as the American Medical Association (AMA). These new procedures are then often initially given provisional codes with limited reimbursement before being transitioned to a permanent reimbursable code. For the AMA system, only about 30% of provisional codes (category III) reach permanent code status [46].

Throughout this process, research funding is vital for generating the data necessary for acceptance by the medical community and medical regulatory entities. However, there may be a disconnect as well-funded technologies with positive evidence must undergo a separate process for insurance coverage that is not necessarily associated with the level of evidence [45]. Some processes exist to help bridge this gap and provide conditional coverage as new techniques await insurance coverage, such as Coverage with Evidence Development (CED) which provides coverage during approved clinical trials or Investigational Device Exemption (IDE) coverage for certain studies [47,48]. However, there remain considerable challenges in the implementation of new surgical technology beyond establishment of a body of research evidence.

### 4.6. Limitations

Our study is not without limitations. As a single-institution retrospective review, there is potential for selection bias of procedures, especially in selecting patients for acute TMR, and inconsistencies in medical records, particularly with our institution transitioning between electronic medical records within the study period. In addition, there was a lack of patient characteristics such as demographic and perioperative or postoperative variables that could have been used for subgroup analysis. These data also relied on CPT coding, which can often be inconsistent or incomplete, leading to procedures being inappropriately included or excluded.

## 5. Conclusions

Our retrospective, single-institutional study from 2002 to 2025 demonstrated increasing utilization of targeted muscle reinnervation for use in post-amputation pain as well as neuropathic pain. Specifically, the paradigm shift from exclusively being used for advanced myoelectric prosthesis control to pain prophylaxis or treatment regardless of major limb loss created numerous downstream effects in practice patterns as shown. Bibliographic analysis showed increasing research activity for TMR, reflecting trends observed in our institutional data. Future work must more effectively identify high-risk patients undergoing major amputation for focused collaborative efforts to provide acute TMR and downstream referral networks for myoelectric prosthesis fitting.

## Figures and Tables

**Figure 1:**
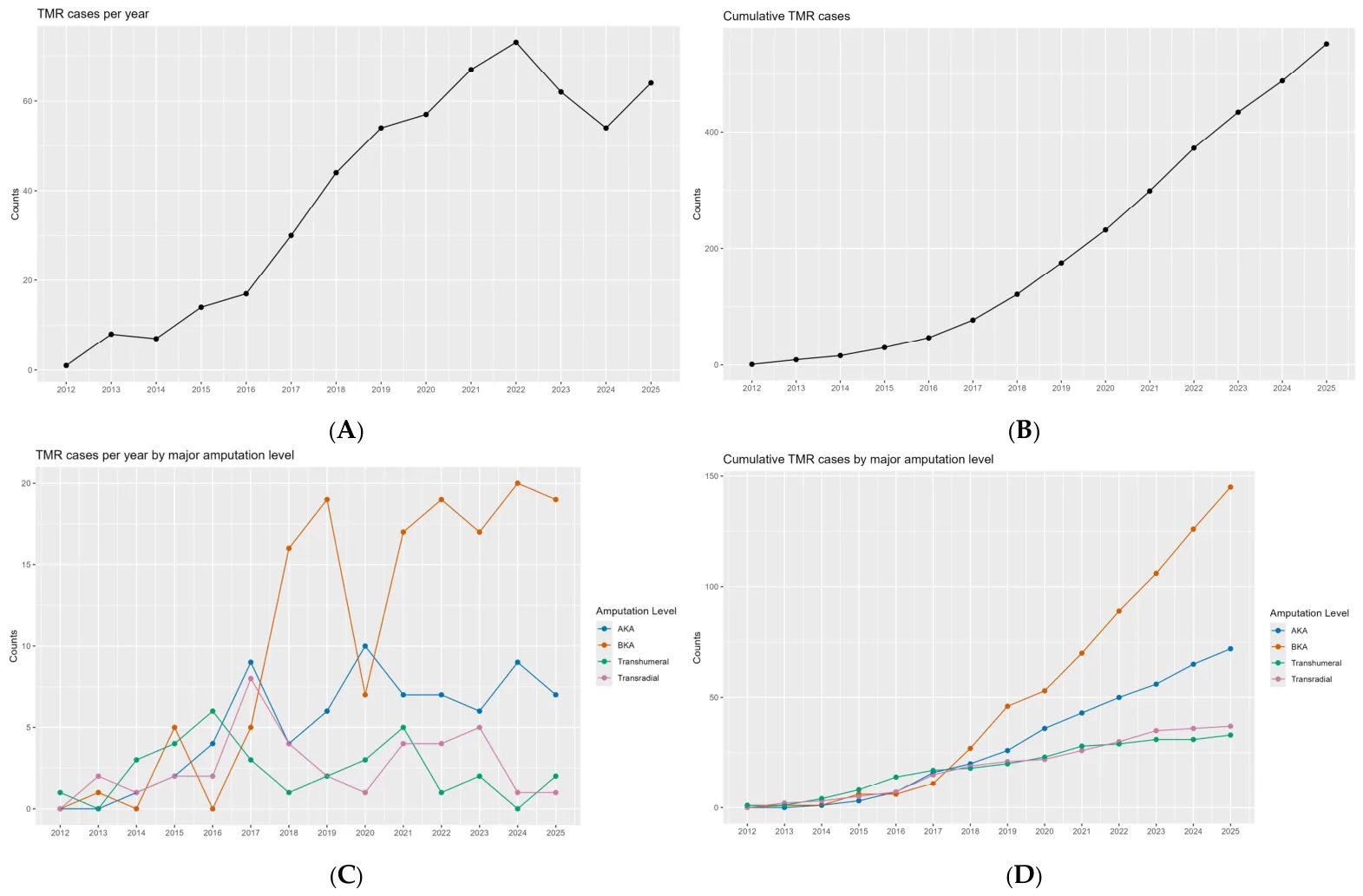
Total TMR volume is presented as annualized (**A**) and cumulative values (**B**). TMR cases by major amputation level are also presented as annualized (**C**) and cumulative values (**D**).

**Figure 2:**
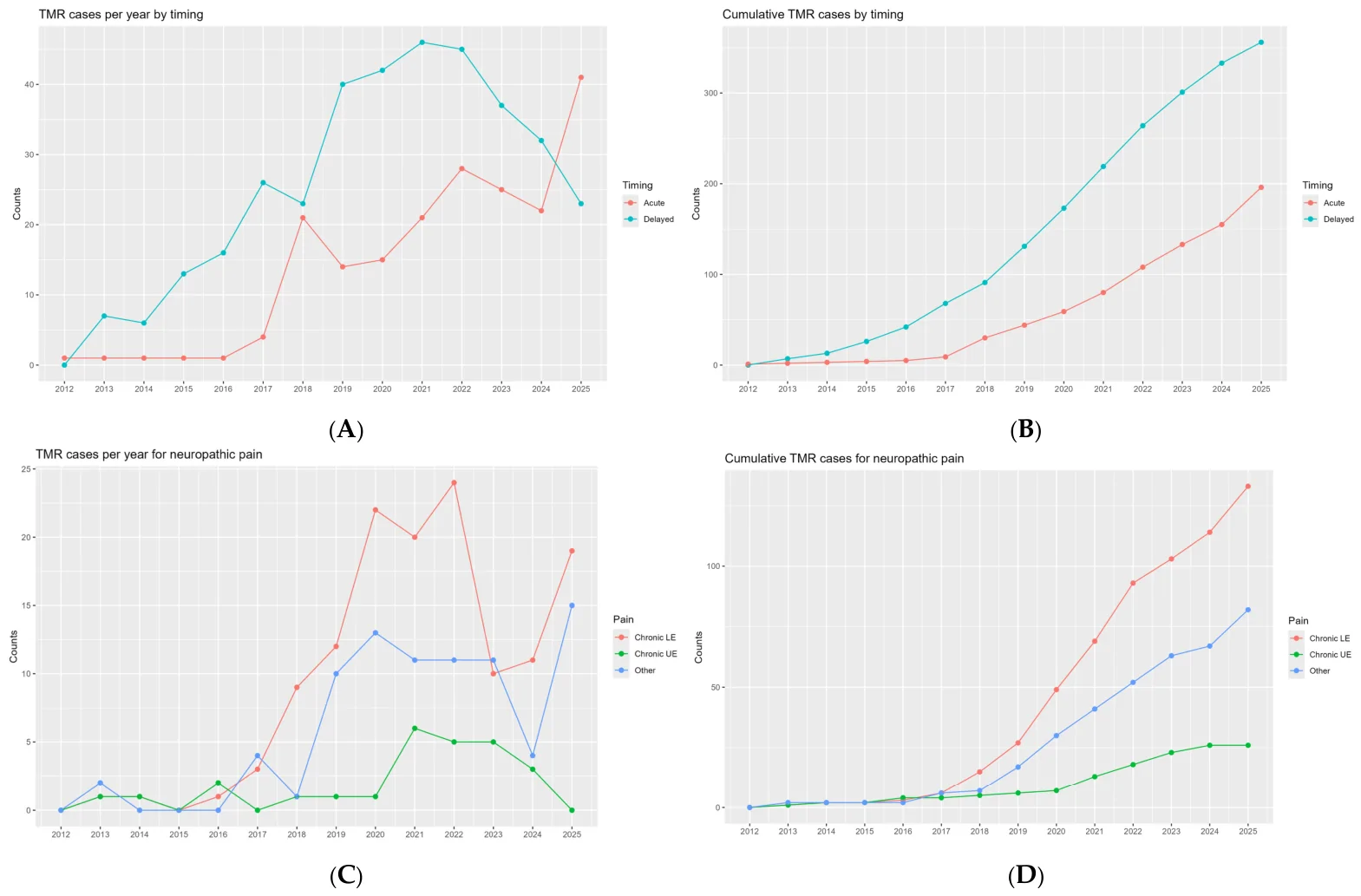
TMR cases by timing of intervention is presented as annualized (**A**) and cumulative values (**B**). TMR cases for neuropathic pain are also presented as annualized (**C**) and cumulative values (**D**).

**Figure 3:**
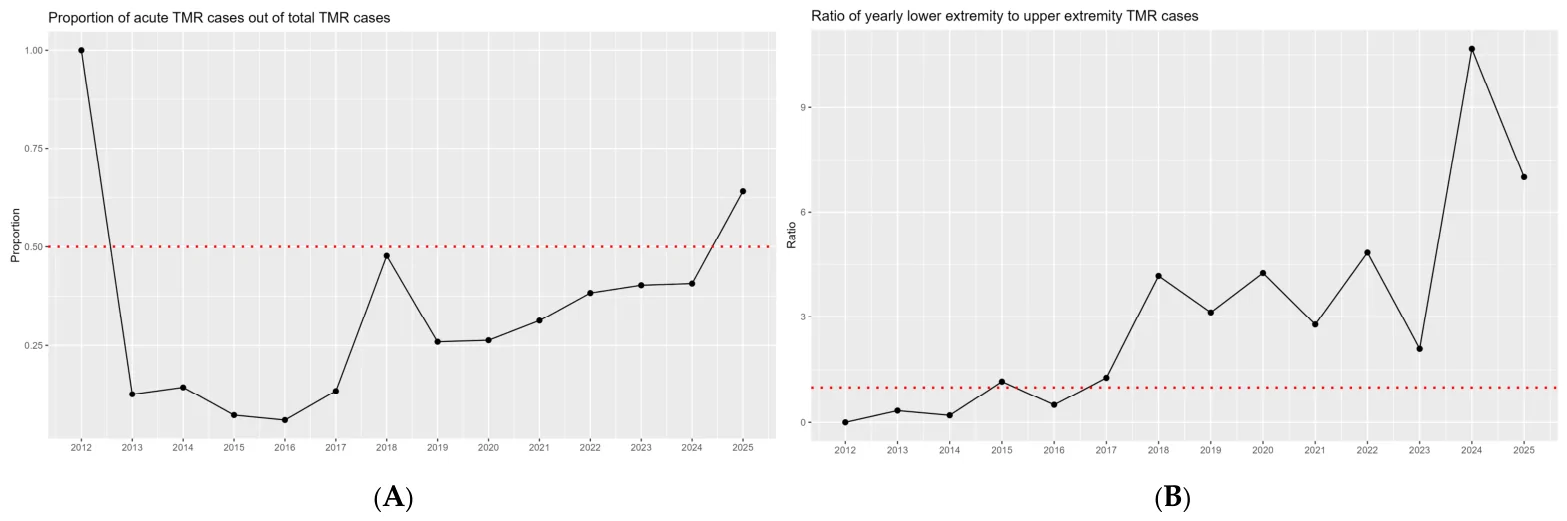
Ratio of TMR cases by timing and extremity are presented. Red dotted line represents a ratio of 0.50 in (**A**) and 1.0 in (**B**).

**Figure 4:**
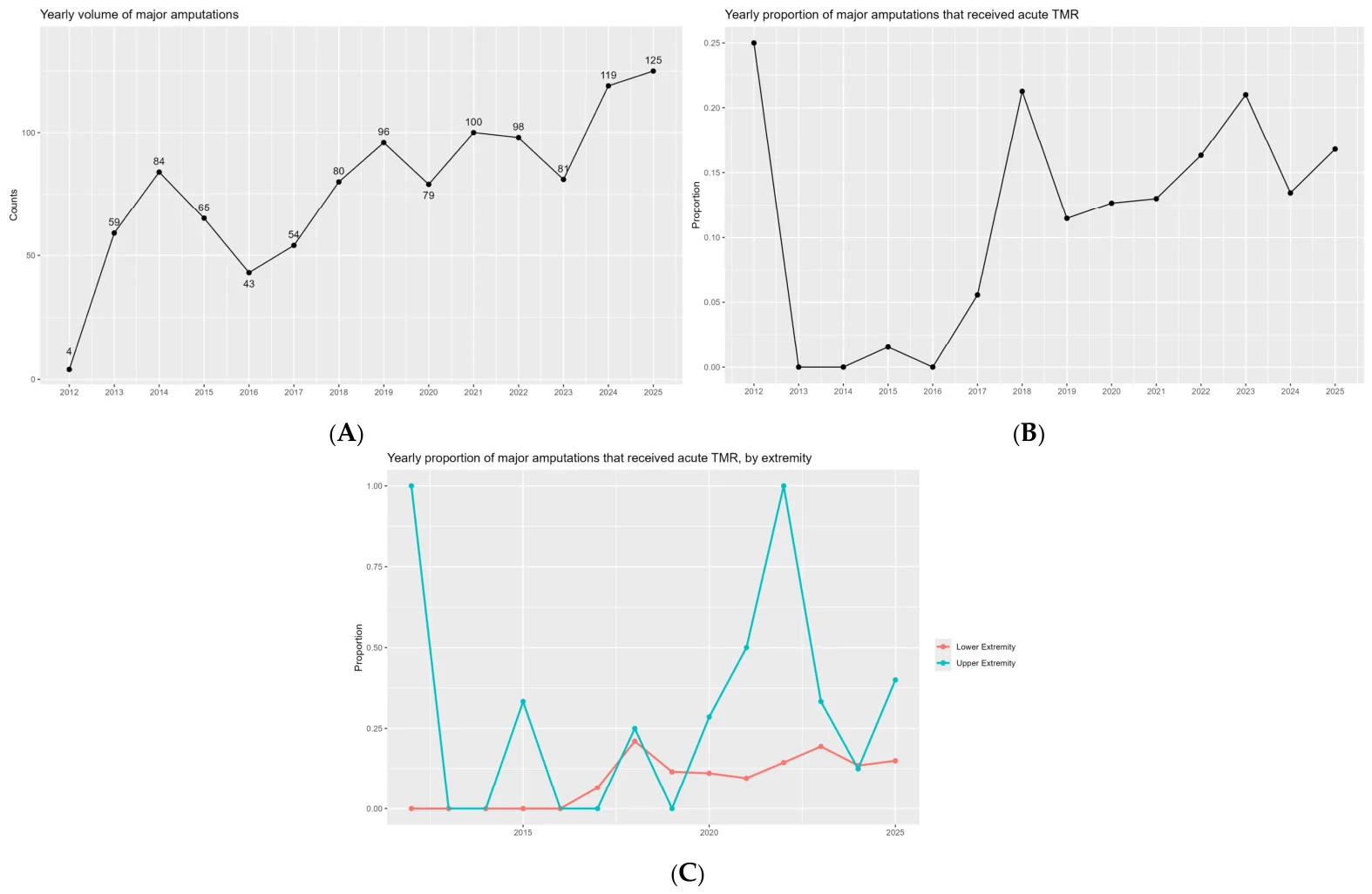
Institutional volume for major amputations per year (**A**) compared to acute TMR volume (**B**,**C**).

**Figure 5:**
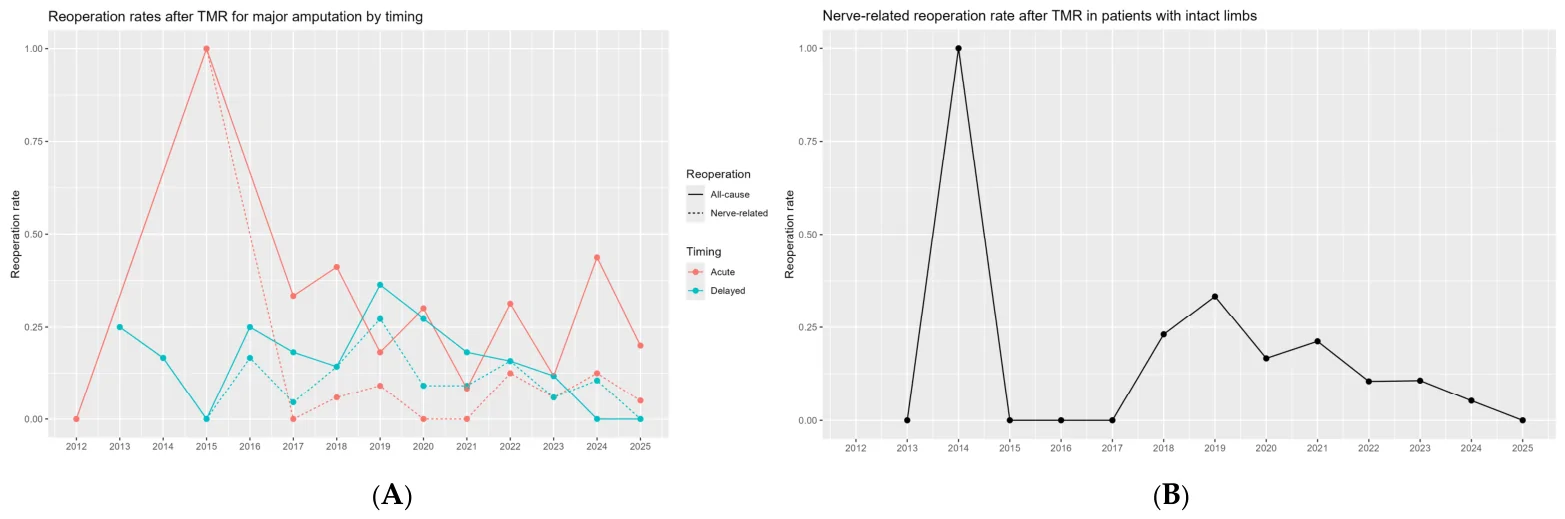
Reoperation rates per year relative to year the index TMR case was performed. (**A**) All-cause (same limb) and nerve-related reoperation rate following acute and delayed TMR for major amputation; (**B**) nerve-related reoperation following TMR in patients with intact limbs.

**Figure 6:**
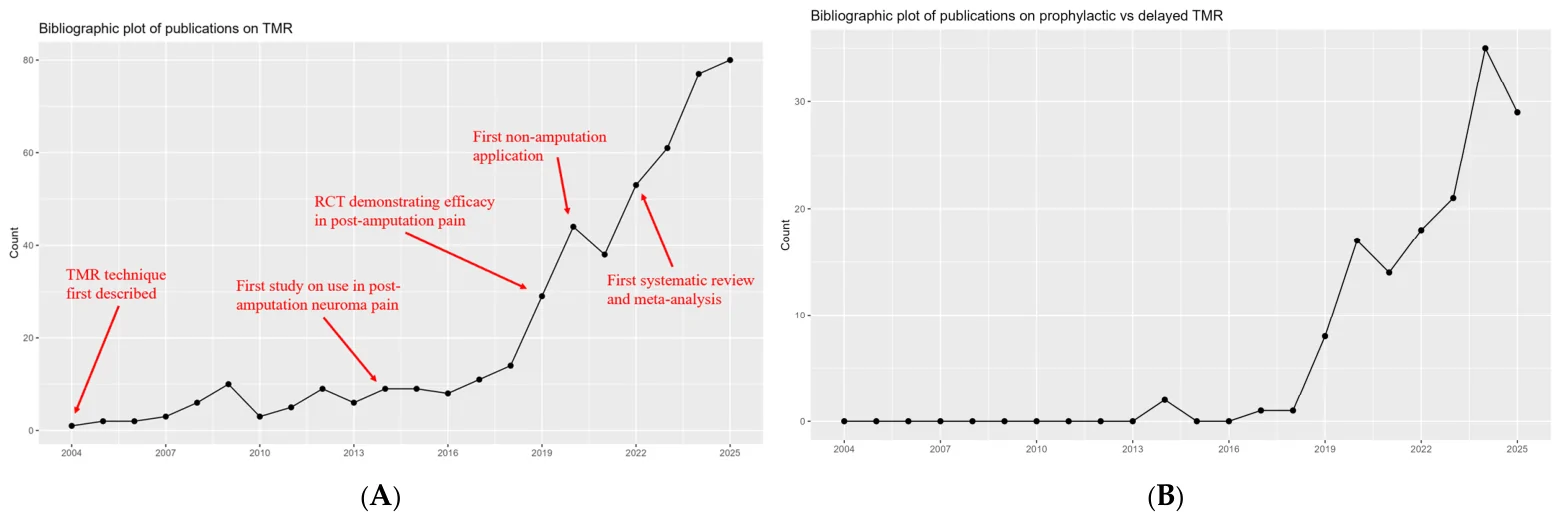

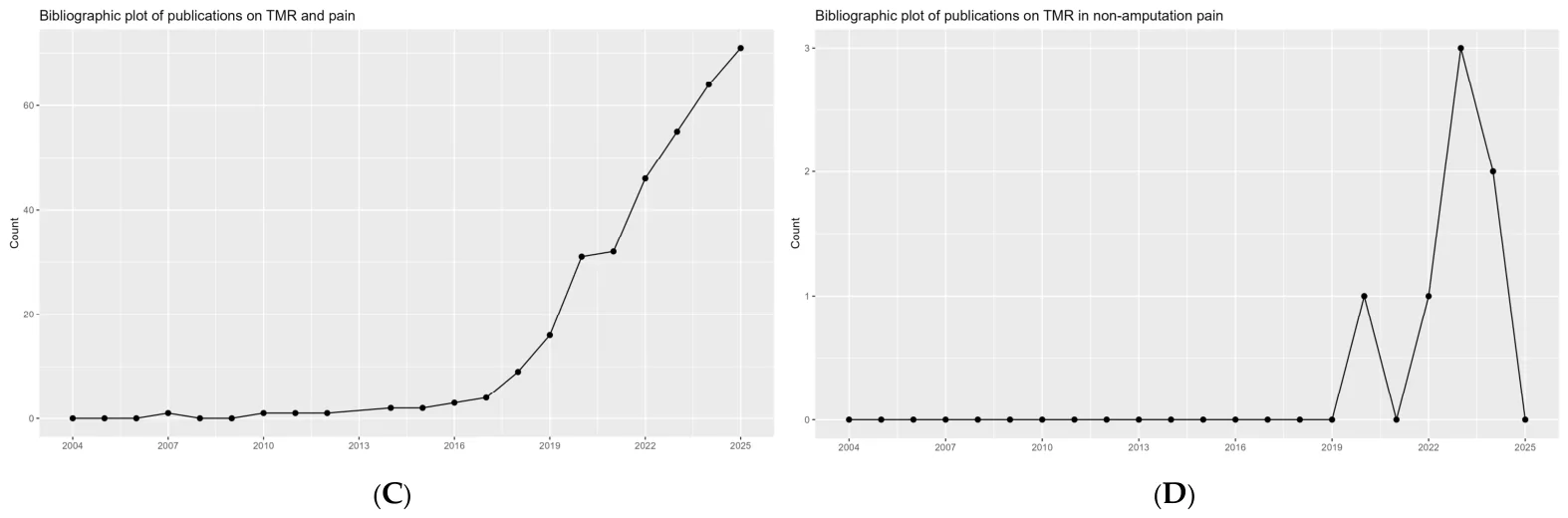
The bibliographic analysis for total TMR publications with annotated milestones (**A**), timing (**B**), pain (**C**), and for non-amputation indications (**D**).

**Table 1:** All-cause and nerve-specific reoperation data for patients with and without limb loss.

Group	Characteristic	Overall N = 316 ^1^	Acute N = 124 ^1^	Delayed N = 192 ^1^	*p* ^2^
Reoperation	All-cause reoperation	64 (20.3%)	33 (26.6%)	31 (16.1%)	0.031
	Reoperations per reoperated patient, median (IQR)	1 (1–2)	1 (1–3)	1 (1–2)	0.075
Reoperation category	Wound-related (any)	32 (10.1%)	23 (18.5%)	9 (4.7%)	<0.001
	Infection	28 (8.9%)	20 (16.1%)	8 (4.2%)	0.002
	Nonhealing wound/ulcer	17 (5.4%)	14 (11.3%)	3 (1.6%)	0.002
	Wound dehiscence	7 (2.2%)	7 (5.6%)	0 (0.0%)	0.004
	Hematoma/seroma	6 (1.9%)	6 (4.8%)	0 (0.0%)	0.007
	Soft tissue/amputation revision	44 (13.9%)	29 (23.4%)	15 (7.8%)	0.001
	Nerve-related	31 (9.8%)	9 (7.3%)	22 (11.5%)	0.250
Reoperation by extremity	All-cause, upper extremity	12/85 (14%)	3/16 (19%)	9/69 (13%)	0.690
	All-cause, lower extremity	52/231 (23%)	30/108 (28%)	22/123 (18%)	0.083
	*p*, upper vs. lower extremity	0.115	0.556	0.421	
	Nerve-related, upper extremity	10/85 (12%)	2/16 (13%)	8/69 (12%)	1.000
	Nerve-related, lower extremity	21/231 (9%)	7/108 (6%)	14/123 (11%)	0.253
	*p*, upper vs. lower extremity	0.523	0.327	1.000	
All-cause reoperation by period of index TMR	2012–2014	2/11 (18%)	0/1 (0%)	2/10 (20%)	1.000
	2015–2020	34/135 (25%)	14/42 (33%)	20/93 (22%)	0.198
	2021–2025	28/170 (16%)	19/81 (23%)	9/89 (10%)	0.023
Among patients with all-cause reoperation	Traumatic indication	6/64 (9%)	1/33 (3%)	5/31 (16%)	0.099

^1^ n (%); Median (Q1–Q3). ^2^ Fisher’s exact test; Wilcoxon rank sum exact test.

## Data Availability

All data can be made available on reasonable request to the corresponding author.

## Supplementary Materials

The following supporting information can be downloaded at: https://www.mdpi.com/article/10.3390/jcm15207874/s1. Table S1. CPT codes utilized for analysis. Table S2. PubMed search queries for bibliographic analysis of articles on TMR.

## References

[1] Hergert B.C., Rana A.-N., Velasquez J.E., Johnson A.E., Ali S., Wong K.A., Teixeira P.G. (2024). Posttraumatic Amputations Epidemiology and Outcomes Within the National Trauma Data Bank: Improved Survival over Time Results in Increased Population in Need of Rehabilitation Support. Am. J. Phys. Med. Rehabil..

[2] Creager M.A., Matsushita K., Arya S., Beckman J.A., Duval S., Goodney P.P., Gutierrez J.A.T., Kaufman J.A., Joynt Maddox K.E., Pollak A.W. (2021). Reducing Nontraumatic Lower-Extremity Amputations by 20% by 2030: Time to Get to Our Feet: A Policy Statement from the American Heart Association. Circulation.

[3] Potter C.B.K., Bosse M.J. (2021). American Academy of Orthopaedic Surgeons Clinical Practice Guideline Summary for Limb Salvage or Early Amputation. J. Am. Acad. Orthop. Surg..

[4] Limakatso K., Bedwell G.J., Madden V.J., Parker R. (2020). The prevalence and risk factors for phantom limb pain in people with amputations: A systematic review and meta-analysis. PLoS ONE.

[5] List E.B., Krijgh D.D., Martin E., Coert J.H. (2021). Prevalence of residual limb pain and symptomatic neuromas after lower extremity amputation: A systematic review and meta-analysis. Pain.

[6] Oosterhoff M., Geertzen J.H.B., Dijkstra P.U. (2022). More than half of persons with lower limb amputation suffer from chronic back pain or residual limb pain: A systematic review with meta-analysis. Disabil. Rehabil..

[7] Stankevicius A., Wallwork S.B., Summers S.J., Hordacre B., Stanton T.R. (2021). Prevalence and incidence of phantom limb pain, phantom limb sensations and telescoping in amputees: A systematic rapid review. Eur. J. Pain.

[8] Biddiss E., Chau T. (2007). Upper-Limb Prosthetics: Critical Factors in Device Abandonment. Am. J. Phys. Med. Rehabil..

[9] Kuiken T.A., Dumanian G.A., Lipschutz R.D., Miller L.A., Stubblefield K.A. (2004). The use of targeted muscle reinnervation for improved myoelectric prosthesis control in a bilateral shoulder disarticulation amputee. Prosthet. Orthot. Int..

[10] Souza J.M., Cheesborough J.E., Ko J.H., Cho M.S., Kuiken T.A., Dumanian G.A. (2014). Targeted muscle reinnervation: A novel approach to postamputation neuroma pain. Clin. Orthop. Relat. Res..

[11] Dumanian G.A., Potter B.K., Mioton L.M., Ko J.H., Cheesborough J.E., Souza J.M., Ertl W.J., Tintle S.M., Nanos G.P., Valerio I.L. (2019). Targeted Muscle Reinnervation Treats Neuroma and Phantom Pain in Major Limb Amputees: A Randomized Clinical Trial. Ann. Surg..

[12] Valerio I.L., Dumanian G.A., Jordan S.W., Mioton L.M., Bowen J.B., West J.M., Porter K., Ko J.H., Souza J.M., Potter B.K. (2019). Preemptive Treatment of Phantom and Residual Limb Pain with Targeted Muscle Reinnervation at the Time of Major Limb Amputation. J. Am. Coll. Surg..

[13] Mauch J.T., Kao D.S., Friedly J.L., Liu Y. (2023). Targeted muscle reinnervation and regenerative peripheral nerve interfaces for pain prophylaxis and treatment: A systematic review. PM&R.

[14] Lanier S.T., Jordan S.W., Ko J.H., Dumanian G.A. (2020). Targeted Muscle Reinnervation as a Solution for Nerve Pain. Plast. Reconstr. Surg..

[15] Johnson C.C., Loeffler B.J., Gaston R.G. (2021). Targeted Muscle Reinnervation: A Paradigm Shift for Neuroma Management and Improved Prosthesis Control in Major Limb Amputees. J. Am. Acad. Orthop. Surg..

[16] Gardner W., Mulvey E.P., Shaw E.C. (1995). Regression analyses of counts and rates: Poisson, overdispersed Poisson, and negative binomial models. Psychol. Bull..

[17] Nick T.G., Campbell K.M. (2007). Logistic regression. Methods Mol. Biol..

[18] The Comprehensive R Archive Network. https://cran.r-project.org/.

[19] Roubaud M.J., Babu A., Moon B.S., Lewis V.O. (2026). Targeted Muscle Reinnervation and Regenerative Peripheral Nerve Interface: The Evolving Landscape in the Treatment of Postamputation Pain and Prosthetics. J. Am. Acad. Orthop. Surg..

[20] Rivera J.A., Churovich K., Anderson A.B., Potter B.K. (2024). Estimating Recent US Limb Loss Prevalence and Updating Future Projections. Arch. Rehabil. Res. Clin. Transl..

[21] Reilly M.A., Cohen I.C., Watson S.L., Minc S.D., Ho K.J., Feinglass J. (2025). A population health analysis of trends in lower extremity amputation secondary to diabetes and peripheral artery disease, 2016–2023. Diabetes Res. Clin. Pract..

[22] Sharath S.E., Natarajan S., Sihaloho D., Ferguson C., Medvedovsky S., Joseph T., Kougias P. (2026). Shifting burden of major lower-extremity amputations across hospital Medicaid burden and socioeconomic groups, 1993 to 2021. J. Vasc. Surg..

[23] ElAbd R., Dow T., Jabori S., Alhalabi B., Lin S.J., Dowlatshahi S. (2024). Pain and Functional Outcomes following Targeted Muscle Reinnervation: A Systematic Review. Plast. Reconstr. Surg..

[24] Bishay J., Yeap I., Wang T. (2024). The effectiveness of targeted muscle reinnervation in reducing pain and improving quality of life for patients following lower limb amputation. J. Plast. Reconstr. Aesthetic Surg. JPRAS.

[25] Fowler X.P., Krafcik B., Cai M., Gladders B., Spangler E., Moore K.O., Wong S., Stone D., Soybel D., Columbo J. (2024). A Retrospective Analysis of Surgical Acuity & Procedure Volume Before & During the COVID-19 Pandemic. J. Surg. Res..

[26] Krueger C.A., Wenke J.C., Ficke J.R. (2012). Ten years at war: Comprehensive analysis of amputation trends. J. Trauma Acute Care Surg..

[27] Chopra A., Azarbal A.F., Jung E., Abraham C.Z., Liem T.K., Landry G.J., Moneta G.L., Mitchell E.L. (2018). Ambulation and functional outcome after major lower extremity amputation. J. Vasc. Surg..

[28] Johannesson A., Larsson G.-U., Ramstrand N., Turkiewicz A., Wiréhn A.-B., Atroshi I. (2009). Incidence of Lower-Limb Amputation in the Diabetic and Nondiabetic General Population. Diabetes Care.

[29] Shutze W., Gable D., Ogola G., Eidt J. (2024). Prosthetic outcomes after amputation and the impact of mobility level on survival. J. Vasc. Surg..

[30] Janes L.E., Fracol M.E., Ko J.H., Dumanian G.A. (2020). Management of Unreconstructable Saphenous Nerve Injury with Targeted Muscle Reinnervation. Plast. Reconstr. Surg.—Glob. Open.

[31] Chappell A.G., Yang C.S., Dumanian G.A. (2021). Surgical Treatment of Abdominal Wall Neuromas. Plast. Reconstr. Surg.—Glob. Open.

[32] Fracol M.E., Dumanian G.A., Janes L.E., Bai J., Ko J.H. (2020). Management of Sural Nerve Neuromas with Targeted Muscle Reinnervation. Plast. Reconstr. Surg.—Glob. Open.

[33] Moradian S., Taritsa I.C., Sharma S., Mioton L., Dumanian G.A., Ko J.H. (2024). Management of Superficial and Deep Peroneal Nerve Neuromas with Targeted Muscle Reinnervation in Nonamputees: Operative Technique and Early Outcomes. Plast. Reconstr. Surg.—Glob. Open.

[34] Li A.T., Garcia N., Angliss M., Paul E., Gray S., Bruscino-Raiola F. (2024). Acute versus non-acute targeted muscle reinnervation for pain control following major limb amputation: A comparative study. J. Plast. Reconstr. Aesthetic Surg. JPRAS.

[35] Goodyear E.G., O’Brien A.L., West J.M., Huayllani M.T., Huffman A.C., Souza J.M., Schulz S.A., Moore A.M. (2024). Targeted Muscle Reinnervation at the Time of Amputation Decreases Recurrent Symptomatic Neuroma Formation. Plast. Reconstr. Surg..

[36] Eberlin K.R., Brown D.A., Gaston R.G., Kleiber G.M., Ko J.H., Kovach S.J., Loeffler B.J., MacKay B.J., Potter B.K., Roubaud M.S. (2023). A Consensus Approach for Targeted Muscle Reinnervation in Amputees. Plast. Reconstr. Surg.—Glob. Open.

[37] Maradit Kremers H., Salduz A., Schleck C.D., Larson D.R., Berry D.J., Lewallen D.G. (2017). Referral Bias in Primary Total Knee Arthroplasty: Retrospective Analysis of 22,614 Surgeries in a Tertiary Referral Center. J. Arthroplast..

[38] Lee K.K.-I., Sadeh O., Barrientos A., Genzelev A., Ayalon O., Agrawal N.A., Bekisz J.M., Hacquebord J.H. (2026). Comparison of Reoperation and Complication Rates Between Acute and Delayed Advanced Nerve Interface Procedures in Lower-Extremity Amputees. J. Clin. Med..

[39] Lans J., Groot O.Q., Hazewinkel M.H.J., Kaiser P.B., Lozano-Calderón S.A., Heng M., Valerio I.L., Eberlin K.R. (2022). Factors Related to Neuropathic Pain following Lower Extremity Amputation. Plast. Reconstr. Surg..

[40] Lans J., Hoftiezer Y., Lozano-Calderón S.A., Heng M., Valerio I.L., Eberlin K.R. (2021). Risk Factors for Neuropathic Pain Following Major Upper Extremity Amputation. J. Reconstr. Microsurg..

[41] Chang B.L., Mondshine J., Attinger C.E., Kleiber G.M. (2021). Targeted Muscle Reinnervation Improves Pain and Ambulation Outcomes in Highly Comorbid Amputees. Plast. Reconstr. Surg..

[42] Shammas R.L., Azoury S.C., Sergesketter A.R., Lee H.-J., Poehlein E., Othman S.E., Cason R.W., Levinson H., Kovach S.J., Mithani S.K. (2022). Primary Targeted Muscle Reinnervation after Below-Knee Amputation Is Not Associated with an Increased Risk of Major or Minor Surgical Complications: A Multi-Institutional, Propensity Score–Matched Analysis. Plast. Reconstr. Surg..

[43] Berger L.E., Shin S., Haffner Z.K., Huffman S.S., Spoer D.L., Sayyed A.A., Franzoni G., Bekeny J.C., Attinger C.E., Kleiber G.M. (2023). The application of targeted muscle reinnervation in lower extremity amputations: A systematic review. Microsurgery.

[44] Simon A.M., Turner K.L., Miller L.A., Dumanian G.A., Potter B.K., Beachler M.D., Hargrove L.J., Kuiken T.A. (2023). Myoelectric prosthesis hand grasp control following targeted muscle reinnervation in individuals with transradial amputation. PLoS ONE.

[45] Sexton Z.A., Perl J.R., Saul H.R., Trotsyuk A.A., Pietzsch J.B., Ruggles S.W., Nikolov M.C., Schulman K.A., Makower J. (2023). Time from Authorization by the US Food and Drug Administration to Medicare Coverage for Novel Technologies. JAMA Health Forum.

[46] Dranove D., Garthwaite C., Heard C., Wu B. (2022). The economics of medical procedure innovation. J. Health Econ..

[47] Mooghali M., Moneer O., Janda G., Dhruva S.S., Ross J.S., Ramachandran R. (2025). Assessing Medicare’s Coverage with Evidence Development Program. Health Aff..

[48] Abbasi A.B., Curtis L.H., Fleisher L.A., Califf R.M. (2024). Why Evidence Generation Should Matter to Payers and How They Can Help. JAMA.

