# Supplementary material for: The Evolution of TMR Utilization: A 23-Year Journey from Exploratory Upper Extremity Prosthesis Control to Standard of Care for Major Amputations and Pain Management
Source: J Clin Med. 2026 Oct 13;15(20):7874. doi: 10.3390/jcm15207874 (PMC13706117; doi:10.3390/jcm15207874)
Supplement: Supplementary file 1 [file jcm-15-07874-s001.zip › jcm-4534375-supplementary.pdf]

Supplemental Table S1. CPT codes utilized for analysis.

**TMR**	64905: nerve pedicle transfer; first stage
**Major amputation levels**	**Transhumeral**<br>24900: amputation, arm through humerus; with primary closure<br>24920: amputation, arm through humerus; open, circular (guillotine)<br>24930: transhumeral re-amputation<br>24931: transhumeral amputation with implant<br><br>**Transradial**<br>25900: amputation, forearm, through radius and ulna<br>25905: amputation, forearm, through radius and ulna; open, circular (guillotine)<br>25909: amputation, forearm, through radius and ulna; reamputation<br><br>**BKA**<br>27880: amputation, leg, through tibia and fibula<br>27881: amputation, leg, through tibia and fibula; with immediate fitting of technique including first cast<br>27882: amputation, leg, through tibia and fibula; open, circular (guillotine)<br>27886: amputation, leg, through tibia and fibula; re-amputation<br>27888: amputation, leg, through tibia and fibula; radical resection for tumor<br><br>**AKA**<br>27590: Amputation, thigh, through femur, any level<br>27591: Amputation, thigh, through femur, any level; immediate fitting technique including first cast<br>27592: Amputation, thigh, through femur, any level; open, circular (guillotine)<br>27596: Amputation, thigh, through femur, any level; re-amputation<br>27598: Disarticulation at knee

Supplemental Table S2. PubMed search queries for bibliographic analysis of articles on TMR.

**Overall**	("targeted muscle reinnervation"[MeSH Terms] OR "targeted muscle reinnervation"[Title/Abstract] OR "targeted reinnervation"[Title/Abstract])
**Amputation**	("targeted muscle reinnervation"[MeSH Terms] OR "targeted muscle reinnervation"[Title/Abstract] OR "targeted reinnervation"[Title/Abstract]) AND ("amputation"[MeSH Terms] OR "amputation"[Title/Abstract] OR "amputee"[Title/Abstract] OR "amputees"[Title/Abstract] OR "limb loss"[Title/Abstract] OR "transhumeral"[Title/Abstract] OR "transradial"[Title/Abstract] OR "transtibial"[Title/Abstract] OR "transfemoral"[Title/Abstract] OR "above-knee amputation"[Title/Abstract] OR "below-knee amputation"[Title/Abstract] OR "above-elbow amputation"[Title/Abstract] OR "below-elbow amputation"[Title/Abstract] OR "shoulder disarticulation"[Title/Abstract] OR "hip disarticulation"[Title/Abstract] OR "residual limb"[Title/Abstract] OR "stump"[Title/Abstract])
**Pain**	("targeted muscle reinnervation"[MeSH Terms] OR "targeted muscle reinnervation"[Title/Abstract] OR "targeted reinnervation"[Title/Abstract]) AND (pain OR "phantom limb pain" OR "phantom pain" OR "residual limb pain" OR "stump pain" OR "postamputation pain" OR "post-amputation pain" OR "amputation pain" OR "neuroma pain" OR "symptomatic neuroma" OR "painful neuroma" OR "end-neuroma" OR "terminal neuroma" OR neuroma OR "neuropathic pain" OR "chronic pain" OR "nerve pain" OR "neurogenic pain" OR neuralgia OR "pain management" OR "pain prevention" OR "pain prophylaxis" OR "pain reduction" OR "pain relief" OR "pain outcome" OR "pain intensity" OR "pain interference" OR "opioid use" OR analgesic)
**Prophylaxis/timing**	("targeted muscle reinnervation"[MeSH Terms] OR "targeted muscle reinnervation"[Title/Abstract] OR "targeted reinnervation"[Title/Abstract]) AND (acute OR immediate OR "at the time of amputation" OR delayed OR secondary OR

	"non-acute" OR timing) AND (pain OR neuroma OR "phantom limb")
**<u>Non-amputation pain</u>**	("targeted muscle reinnervation"[MeSH Terms] OR "targeted muscle reinnervation"[Title/Abstract] OR "targeted reinnervation"[Title/Abstract]) AND ("occipital neuroma"[Title/Abstract] OR "occipital nerve"[Title/Abstract] OR "greater occipital nerve"[Title/Abstract] OR "lesser occipital nerve"[Title/Abstract] OR "third occipital nerve"[Title/Abstract] OR "occipital neuralgia"[Title/Abstract] OR "occipital nerve entrapment"[Title/Abstract] OR "occipital nerve injury"[Title/Abstract] OR "occipital neuropathy"[Title/Abstract] OR "C2 neuralgia"[Title/Abstract] OR "posterior scalp neuroma"[Title/Abstract] OR "cranial neuroma"[Title/Abstract] OR "post-craniotomy neuroma"[Title/Abstract] OR "post-craniotomy pain"[Title/Abstract] OR "suboccipital neuroma"[Title/Abstract] OR "intercostal neuroma"[Title/Abstract] OR "chest wall neuroma"[Title/Abstract] OR "thoracic neuroma"[Title/Abstract] OR "thoracic wall pain"[Title/Abstract] OR "post-thoracotomy neuroma"[Title/Abstract] OR "post-thoracotomy pain"[Title/Abstract] OR "post-mastectomy neuroma"[Title/Abstract] OR "post-mastectomy pain"[Title/Abstract] OR "post-sternotomy neuroma"[Title/Abstract] OR "post-sternotomy pain"[Title/Abstract] OR "rib neuroma"[Title/Abstract] OR "thoracic nerve injury"[Title/Abstract] OR "anterior cutaneous nerve entrapment"[Title/Abstract] OR "ACNES"[Title/Abstract] OR "lateral cutaneous nerve"[Title/Abstract] OR "post-surgical chest wall pain"[Title/Abstract] OR "groin neuroma"[Title/Abstract] OR "inguinal neuroma"[Title/Abstract] OR "ilioinguinal neuroma"[Title/Abstract] OR "ilioinguinal nerve"[Title/Abstract] OR "iliohypogastric neuroma"[Title/Abstract] OR "iliohypogastric nerve"[Title/Abstract] OR "genitofemoral neuroma"[Title/Abstract] OR "genitofemoral nerve"[Title/Abstract] OR "inguinal

	neuralgia"[Title/Abstract] OR "inguinodynia"[Title/Abstract] OR "groin pain"[Title/Abstract] OR "chronic groin pain"[Title/Abstract] OR "post-herniorrhaphy pain"[Title/Abstract] OR "post-herniorrhaphy neuralgia"[Title/Abstract] OR "post-hernia repair pain"[Title/Abstract] OR "post-inguinal hernia neuroma"[Title/Abstract] OR "groin nerve injury"[Title/Abstract] OR "groin nerve entrapment"[Title/Abstract] OR "inguinal nerve entrapment"[Title/Abstract] OR "lateral femoral cutaneous nerve"[Title/Abstract] OR "meralgia paresthetica"[Title/Abstract] OR "pudendal neuroma"[Title/Abstract] OR "obturator nerve neuroma"[Title/Abstract] OR "post-surgical groin pain"[Title/Abstract] OR "chronic post-surgical inguinal pain"[Title/Abstract] OR "truncal neuroma"[Title/Abstract] OR "abdominal wall neuroma"[Title/Abstract] OR "axial neuroma"[Title/Abstract] OR "non-extremity neuroma"[Title/Abstract] OR "head and neck neuroma"[Title/Abstract] OR "scalp neuroma"[Title/Abstract] OR "torso neuroma"[Title/Abstract] OR "pelvic neuroma"[Title/Abstract] OR "perineal neuroma"[Title/Abstract] OR "flank neuroma"[Title/Abstract] OR "scar neuroma"[Title/Abstract] OR "surgical scar neuroma"[Title/Abstract] OR "atypical neuroma"[Title/Abstract] OR "unusual neuroma location"[Title/Abstract])
